# COVID-19 and Autoimmune/Autoinflammatory Rheumatic Disease Patients: Lessons Learned and Questions Anticipating Answers

**DOI:** 10.31138/mjr.32.3.188

**Published:** 2021-09-06

**Authors:** Athanasios-Dimitrios Bakasis, Haralampos M. Moutsopoulos

**Affiliations:** 1 Department of Pathophysiology, School of Medicine, National and Kapodistrian University of Athens, Athens, Greece; 2 Academy of Athens, Athens, Greece

For two years now, the international community is experiencing a pandemic caused by the novel coronavirus (SARS-CoV-2). The clinical picture of the disease, called corona virus disease 19 (COVID-19), is diverse, extending from an asymptomatic infection to a severe life-threating viral syndrome. As early as the beginning of the pandemic, it has been identified that individuals of older age with comorbidities such as obesity, cardiovascular disease, diabetes mellitus, and cancer are at higher risk for experiencing severe and/or fatal COVID-19.^1^ However, the impact of COVID-19 in patients with autoimmune and autoinflammatory rheumatic diseases (AARD) remained unclear.

There has been a surge in molecular biology and bio-technology enabling the deeper understanding of the immunopathogenesis of autoimmune and autoinflammatory rheumatic diseases (AARD). Through this, the identification of molecular targets has led to the development of an array of chemical immunosuppressive/immunomodulatory medications (DNA breaking agents, antimetabolites, NFkβ, JAK-kinase and calcineurin inhibitors) and biologic agents such as inhibitors of pro-inflammatory cytokines, B-lymphocyte growth factors, communication molecules among antigen presenting cells and T-lymphocytes and peripheral blood B-lymphocyte eradica tors. Under these therapies, the auto-hyperactive immune system is “downregulated”, the auto-inflammatory process is ameliorated, and the tissue injury is prevented and possibly reversed. The immunosuppressive/immunomodulatory treatments, however, render the immune system to a compromised state and it might become unable of quelling viral, bacterial, fungal, and parasitic infections^2^. Based on this knowledge, it was hypothesised that AARD patients belong to a high-risk group of individuals for severe and/or fatal COVID-19. Careful monitoring of infected AARD patients with SARS-CoV-2 provided a different picture. In the early months of the pandemic, case reports and case series of AARD patients infected by SARS-CoV-2 revealed that these patients run an asymptomatic, mild or moderate in severity COVID-19.^3,4^ This rather mild course of COVID-19 in AARD patients was attributed, on one hand, to the anti-cytokine therapy they were receiving, which ameliorated the development of the cytokine storm induced by SARS-CoV-2, the fundamental aberrant immune response to the virus in individuals who develop serious and/or fatal COVID-19.^5^ On the other hand, the heightened endogenous type I interferon signature that characterizes autoimmune disease patients^6^ may contribute to early viral clearance and prevent the exaggerated inflammatory responses.^7^

Our group conducted a prospective study exploring COVID-19 manifestations, outcomes, as well as antibody responses to SARS-CoV-2 among infected AARD patients.^8^ It was shown that the majority of patients (68.8%) experienced a mild disease course, about a quarter (23.4%) required inpatient treatment, and the mortality rate was 1.2%. Regarding symptomatology, fatigue, low grade fever, respiratory, and musculoskeletal complaints were predominant in the clinical picture of these patients. High-grade fever, shortness of breath, and confusion were significantly more common among patients requiring hospitalisation compared to the non-hospitalised. Anosmia occurred more frequently in patients recovering at home compared to infected patients requiring hospitalisation. Patients experiencing a more severe disease course were older, had past medical history of lung interstitial pathology in the context of their AARD, and were on therapeutic schemes based on corticosteroids, mycophenolate mofetil, and rituximab. Similar were the findings when the need for hospitalisation was evaluated. AARD flares were an infrequent phenomenon and anti-SARS-CoV-2 antibodies were detectable in 3 out of 4 patients being tested. After the publication of our study, we observed 11 additional AARD patients with COVID-19 from our centers and their clinical course was like the one observed in the published study.

Taking into consideration other studies from around the world with larger number of patients (**Table 1**), it appears that COVID-19 runs a mild-to-moderate in severity disease form in most AARD patients. Predisposing factors for severe disease, hospitalisation, and COVID-19 related death of AARD patients were similar to the ones described in our work.^8–12^ Furthermore, the same therapeutic agents which were described in our study led AARD patients with COVID-19 to severe outcome and death. Mortality of AARD patients from COVID-19 was also found to be comparable to the one in the general population^13^. It is also of interest that AARD patients diseased with COVID-19 did not experience severe exacerbations of their underlying AARD. However, COVID-19 can, in some apparently normal individuals, induce autoimmune diseases such as arthritis, antiphospholipid syndrome, autoimmune cytopenia, and Kawasaki disease.^5^ In addition, many AARD patients do not develop antibodies against SARS-CoV-2 after recovering from COVID-19, partially explained from the treatment regimens they were on. Nevertheless, some medications utilised in the treatment of rheumatic diseases, depending on the timing of administration, may be used as therapeutic strategies against severe COVID-19.^14,15^ Thus, long follow up of AARD patients infected by SARS-CoV-2 is mandatory to examine if they will develop long-term COVID-19 side effects in the future.

**Table 1.** Summary of select published studies assessing COVID-19 course in autoimmune and autoinflammatory rheumatic diseases.

**Reference**	**Objectives**	**Patient Population**	**Outcomes-Conclusions**
COVID-19			
Strangfeld et al.	Factors associated with COVID-19 related death	3729	1) Known general factors (older age, male sex, and specific comorbidities) 2) Disease-specific factors (disease activity and rituximab, sulfasalazine, azathioprine, cyclophosphamide, ciclosporin, mycophenolate or tacrolimus and not receiving any anti-rheumatic drug),
Gianfransesco et al.	Demographic and clinical factors associated with COVID-19 hospitalisation	600	1) Prednisone dose ≥10 mg/day was associated with higher odds of hospitalisation 2) Conventional disease-modifying antirheumatic drug alone or in combination with biologics, Janus kinase inhibitors and antimalarials were not associated with hospitalization 3) Tumour necrosis factor inhibitor use was associated with a reduced odds of hospitalisation
French RMD COVID-19 cohort	Epidemiological characteristics associated with severe disease and death. Mortality in hospitalised patients for COVID-19 with and without rheumatic disease	694	1) 63% developed mild (not hospitalised), 24% moderate (hospitalised out of the ICU) and 13% severe (ICU/deceased) COVID-19 2) Older age, female gender, high body mass index, hypertension and use of corticosteroids, mycophenolate mofetil or rituximab were associated with severe infection 3) Mortality rate was 8% 4) When compared with matched controls, the odds ratio of mortality was 1.45
Marques et al.	Risk factors associated with unfavourable outcomes	334	1) 33.0% were hospitalised, 15.0% were admitted to the ICU and 10.5% underwent mechanical ventilation; 8.4% died 2) Age above 50 years old and immunosuppression with glucocorticoids and cyclophosphamide were associated with unfavourable outcomes of COVID-19

With this virus posing novel challenges and COVID-19 turning to become endemic, vaccination against SARSCoV-2 is of paramount importance to protect AARD patients and achieve herd immunity. The available vaccines so far, demonstrate encouraging safety and efficacy profiles and recent data support long-term durability of vaccine-induced immunity compared to natural infection in the general population.^16^ It is not known, however, for how long AARD patients who suffered from COVID-19 and developed humoral response (antibodies) against SARS-CoV-2 will maintain a sufficient and effective anti-viral immunity. From follow-up studies of COVID-19 infected individuals without AARD, it was shown that the antibodies remain at high levels at least for 5 months post infection.^17^ Sequential studies of the anti-SARS-CoV-2 antibody serum levels are needed, in order to indicate when the optimum time is for AARD patients recovered from COVID-19 to get vaccinated. In the same context, it remains unclear whether AARD patients infected by SARS-CoV-2 who did not mount viral specific humoral immune response, should receive one or two vaccine doses for the development of protective immunity against the virus. Some authorities recommend evaluation of the T-Lymphocyte specific responses, in order to allow vaccination in these patients to strengthen their immune system against SARS-CoV-2. This is not a scientifically-based suggestion, since to our knowledge, there are not, so far, studies showing that COVID-19 patients who did not develop anti-SARS-CoV-2 antibodies have developed anti-viral T-cell immune response. Thus, prioritisation of vaccination should be recommended to this patient group. In addition, it is not known if mixing of mRNA and adenovirus-based vaccines is more effective in this patient population as is in normal people,^18^ as well as if vaccination will be a “one and done” situation, or a booster dose will be needed, given the alarming spreading of new variants.

From a clinical standpoint, it was likely that the myriad of rheumatic diseases and immunosuppressive/immunomodulatory medications might alter COVID-19 vaccine immunogenicity. Firstly, studies have substantiated that treatment with methotrexate, mycophenolate mofetil or rituximab may diminish antibody responses to COVID-19 vaccines.^19^ Secondly, until robust data are generated regarding optimal management of disease-modifying antirheumatic drugs during the vaccination period, we should probably take into consideration precautions similar to that taken with the influenza, pneumococcal, hepatitis, and tetanus vaccines.^20^ Interesting questions arising are whether AARD patients are ultimately benefited from modifications in therapy, taking into consideration the risk for a disease flare, or which parameter should guide the vaccination of AARD patients recently treated with anti-CD20. Is a 6- to 8-month period between the last anti-CD20 administration and vaccination adequate for patients to mount a specific antiviral immunity, or should it be suggested to these patients to get vaccinated when the serum immunoglobulin levels return to normal values? There has been much achieved since the pandemic was declared regarding AARD patients and COVID-19; however, many issues remain to be answered. As Mark Twain once said: *“It ain’t what you don’t know that gets you into trouble. It’s what you know for sure that just ain’t so.”* Additional well-planned and rigorous research in a cohesive and collaborative manner will improve our knowledge and will provide evidence-based answers.
